# Reconstruction of Dorsal Toe Ulcers Using Reverse Dorsal Cross-Toe Adipofascial and Transposition Flap

**DOI:** 10.1055/s-0044-1801789

**Published:** 2025-01-07

**Authors:** B.A. Ramesh, Makam Amruthavalli, Sruthi S., Sathish Kumar J.

**Affiliations:** 1 Department of Plastic Surgery, Sri Ramachandra Medical College and Research Institute, Porur, Chennai, Tamil Nadu, India

**Fig. 1:**
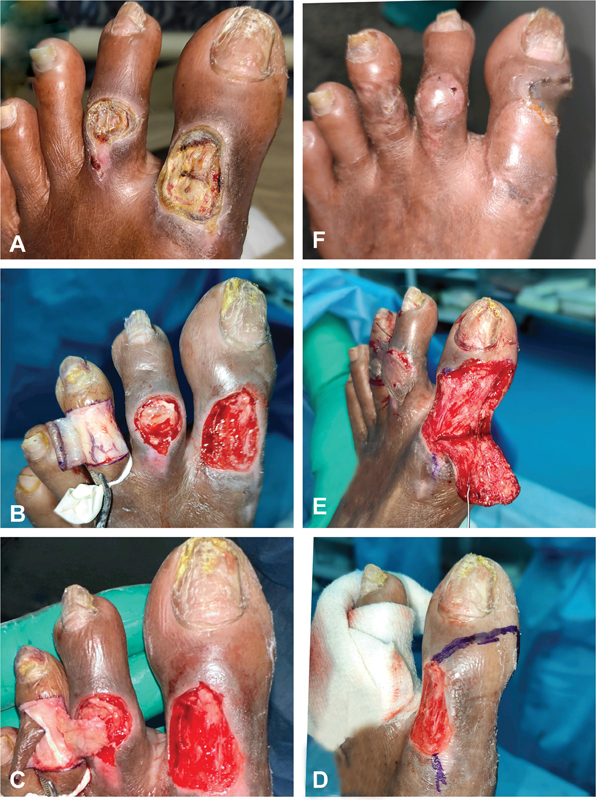
Dorsal toe ulcer reconstruction. ( **A** ) Wounds on the second and great toe dorsum, exposing bone. ( **B** ) Dermal flap elevated under digital tourniquet. ( **C** ) Adipofascial flap elevated above the extensor digitorum tendon. ( **D** ) Markings for the transposition flap on the medial side of the great toe. ( **E** ) Elevation of the transposition flap and split-skin graft (SSG) over the second toe's adipofascial flap. ( **F** ) Healed wounds on both toes.

Dorsal toe skin defects present a significant challenge in reconstructive surgery. This report details the case of a 53-year-old male nonsmoker who presented with two nonhealing ulcers on the dorsum of his left great toe and second toe. The ulcers, which had developed spontaneously, persisted for 2 months despite conservative measures. The patient had a 10-year history of diabetes mellitus and was diagnosed with peripheral arterial disease (PAD), characterized by monophasic flow in the anterior tibial artery and triphasic flow in the posterior tibial artery. On examination, the ulcer on the dorsum of the great toe measured 3 cm × 2.5 cm and exposed the proximal phalanx bone. A second ulcer, measuring 2 cm × 1.5 cm, was located over the proximal interphalangeal joint of the second toe ( Fig. 1A ). The nature of the disease and available treatment options, including fillet flaps or toe amputation, were explained to the patient. However, he strongly desired to preserve his toes for as long as possible, leading us to explore alternative reconstructive strategies. Our vascular surgeons performed lower limb vessel angioplasty, successfully restoring triphasic flow in the anterior tibial artery, thereby improving perfusion to the affected toes.

**Fig. 2:**
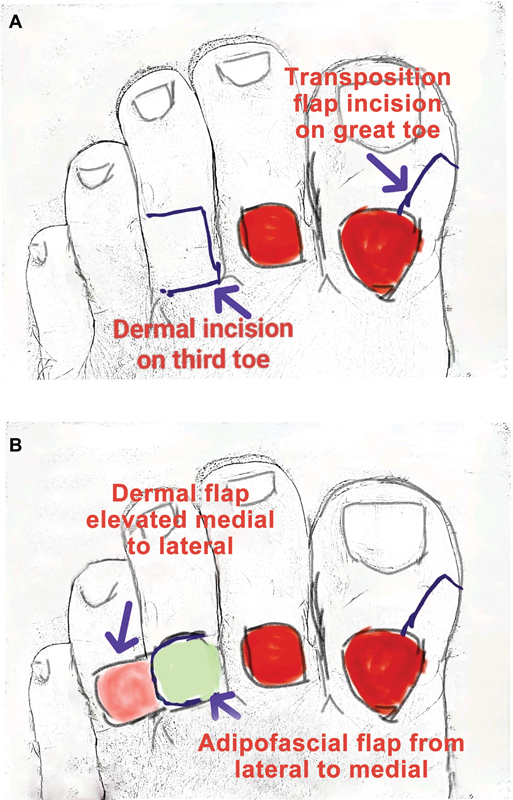
( **A** ) Dermal flap incision on the second toe and transposition flap incision on the great toe. ( **B** ) Dermal flap elevation medial to lateral and adipofascial flap from lateral to medial.

**Fig. 3:**
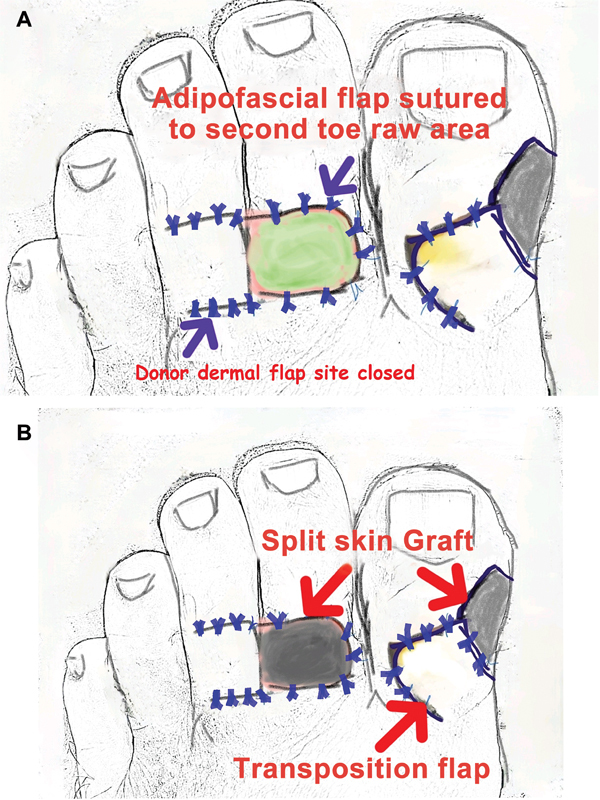
( **A** ) Adipofascial flap and transposition flap sutured. ( **B** ) Skin graft applied to the adipofascial flap and the donor area of the transposition flap.

The ulcers were debrided until healthy bleeding was observed. To address the defect on the second toe, a cross-toe reverse dorsal adipofascial flap was harvested from the third toe. A glove tourniquet was applied to the base of the third toe. Dermal flaps were elevated from medial to lateral ( Fig. 1B ), and the adipofascial flap was elevated from lateral to medial, preserving the paratenon of the extensor digitorum tendon ( Figs. 1C and 2 ). The adipofascial flap was then sutured to the raw area on the second toe using 4–0 absorbable sutures, and a split-thickness skin graft was applied over it. The donor site on the third toe was closed with dermal flaps ( Fig. 3 ). The ulcer on the dorsum of the great toe was covered using a transposition flap from the medial side. The donor site was closed with a split-thickness skin graft ( Fig. 1D, E ). The cross-toe adipofascial flap between the third and second toes was divided 2 weeks later. At follow-up, both the great toe and the second toe wounds demonstrated successful healing ( Fig. 1F ).

The management of PAD through angioplasty was critical in restoring adequate blood flow, which facilitated wound healing. 1 The dorsal and plantar metatarsal arteries primarily supply the toes, and the adipofascial flap technique preserved these vital vascular supplies. Various reconstructive options for dorsal toe defects have been described, including local homo-digital flaps, homo-digital reverse-flow island flaps, and lateral toe pulp flaps. 2 3 4 While the cross-toe adipofascial flap is rarely reported in the literature, the cross-finger adipofascial flap is well documented and effective for dorsal finger defects involving exposed tendons or bones. 5 Although the outcome in this case was satisfactory, toe flaps should be used cautiously in patients with PAD due to the potential risks of compromised perfusion.
